# Female sex is associated with a lower risk of bone metastases and favourable prognosis in non-sex-specific cancers

**DOI:** 10.1186/s12885-019-6168-1

**Published:** 2019-10-25

**Authors:** Wenjuan Ma, Karl Peltzer, Lisha Qi, Guijun Xu, Zheng Liu, Jingyi Wang, Min Mao, Vladimir P. Chekhonin, Xin Wang, Chao Zhang

**Affiliations:** 1 Department of Breast Imaging, Tianjin Medical University Cancer Institute and Hospital, National Clinical Research Center for Cancer, Key Laboratory of Cancer Prevention and Therapy, Tianjin’s Clinical Research Center for Cancer, Tianjin, China; 2 Department of Research & Innovation, University of Limpopo, Turfloop, Mankweng, South Africa; 3 Department of Pathology, Tianjin Medical University Cancer Institute and Hospital, National Clinical Research Center for Cancer, Key Laboratory of Cancer Prevention and Therapy, Tianjin’s Clinical Research Center for Cancer, Tianjin, China; 4 Department of Bone and Soft Tissue Tumors, Tianjin Medical University Cancer Institute and Hospital, National Clinical Research Center for Cancer, Key Laboratory of Cancer Prevention and Therapy, Tianjin’s Clinical Research Center for Cancer, Huanhu Xi Road, Tianjin, 300060 China; 5 Department of Pathology and Southwest Cancer Center, First Affiliated Hospital, Army Medical University, Chongqing, 400038 China; 6 Department of Fundamental and Applied Neurobiology, V. P. Serbsky National Medical Research Center for Psychiatry and Narcology, the Ministry of Health of the Russian Federation, Moscow, Russia; 7 Department of Epidemiology and Biostatistics, First Affiliated Hospital, Army Medical University, 30 Gaotanyan Street, Shapingba District, 400038 China

**Keywords:** SEER, Sex disparity, Bone metastases, Prevalence, Prognosis

## Abstract

**Background:**

The objectives were to investigate the disparity in the prevalence of bone metastases (BM) between the sexes and to assess the effect of female sex on the development and prognosis of BM.

**Methods:**

Cases of invasive non-sex-specific cancers diagnosed between 2010 and 2015 in the Surveillance, Epidemiology, and End Results (SEER) program were used. The prevalence of BM was calculated by combining the prevalence of BM among different cancers. Multivariable logistic regression and proportion hazard regression were conducted to investigate the effect of female sex, and the results were pooled by meta-analysis.

**Results:**

The pooled prevalence of BM among male and female patients was 2.3% (95% CI: 1.6–3.2%) and 1.8% (95% CI: 1.2–2.6%), respectively. The pooled prevalence of BM dramatically decreased for patients aged 11–40 years old, plateaued for patients aged 41–90 years old and increased for patients aged > 90 years old in both male and female patients. Meta-analysis suggested that female sex had a protective effect on the development of BM (pooled OR = 0.80; 95% CI: 0.75–0.84; *p* < .001) and a favourable prognosis for respiratory system cancers (pooled HR = 0.81; 95% CI: 0.71–0.92; *p* < .001). However, no significant associations existed for other cancers. Male non-sex-specific cancer patients and those with male-leaning genetic variations or hormonal status have a greater likelihood of developing BM than female patients.

**Conclusions:**

Female sex was associated with fewer BM in various non-sex-specific cancers, and the effect was constant with changes in age. Female sex showed a protective effect exclusively on the prognosis of respiratory system cancers.

## Background

As previously reported, bone is the third most common metastatic site for malignant cancer [1, 2]. Bone metastases (BM) can cause a series of skeletal-related events (SREs), including pain, bone fractures, spinal cord compression and hypercalcemia [3, 4]. The incidence of SRE has been reported in several studies, the cumulative SRE incidences were respectively reported to be 47, 31.4, and 38.0% in breast cancer, prostate cancer and multiple myeloma patients [5]. Another study reported SRE in 26% patients with prostate cancer, 70% in renal cell carcinoma, and 58% in urothelial carcinoma [6]. Both BM and SRE were thought to negatively impact patient survival. The number of bone metastasis was significantly correlated with the mortality of patients [7, 8]. The latest study showed that the 1-year survival rate of cancer patients with BM ranged from 10% in lung cancer patients to 51% in breast cancer patients [2]. Bone-modifying agents were accepted to significantly retard the first occurrence of SRE [6, 9]. Bisphosphonate was reported to be correlated with the better outcome in patients with bone metastasis [10, 11]. Treatments that prevent SREs have been reported to significantly improve daily function [12].

Accurate estimation of BM is crucial for the prevention and treatment of BM. Previous studies have presented an inconsistent prevalence of BM, which is influenced by various factors [13–17]. It was reported that male sex was one of the independent risk factors for the development of BM [18]. Several studies revealed fewer BM in female patients than in male patients [19–21]. In addition, few studies have investigated the disparity in prognosis between the sexes among patients with BM [22, 23]. The absent consensus regarding the influence of sex on BM was thought to be caused by the small sample size and different characteristics of cancers [17]. Thus, a further study based on a large population is warranted to examine the correlation between sex and the occurrence and prognosis of BM in various cancers.

The National Cancer Institute’s Surveillance, Epidemiology, and End Results (SEER) program was established in 1973 and comprises approximately 30% of the total US population; it is an important data source for epidemiologic analyses. In this study, using the records in the SEER datasets, we aimed to investigate the disparity in the prevalence of BM between the sexes for different non-sex-specific cancers and to evaluate the effect of the female sex on the occurrence and prognosis of BM.

## Methods

### Data source and cohort selection

SEER*Stat version 8.3.5 (Information Management Services, Inc. Calverton, MD) was applied to generate the case listing from the SEER program. The inclusion criteria were as follows: International Classification of Diseases for Oncology, 3rd edition (ICD-O-3) codes for confirmed malignant cancers; diagnosis between 2010 and 2015, as the status of BM was not initially collected by SEER until 2010; and clear information on the bone metastases (yes or no). Patients were excluded if they had a diagnosis of breast cancer or a genital system cancer, such as prostate or ovarian cancer, and if they were diagnosed upon autopsy or via a death certificate.

### Statistical analysis

The prevalence of BM for male and female patients with each cancer type was calculated as the percentage of the subjects with BM within the total number of cancer patients. Moreover, the prevalence of BM for different systems, the total population and the different age groups were calculated by a meta-analysis that combined the prevalence of BM of different cancer types. Multivariable logistic regression was conducted to investigate the effect of female sex on the development of BM in various types of cancer after adjusting for age, race, insurance status, marital status, histological differentiation grade, tumour size, lymphatic metastasis and brain, liver and lung metastasis status. Subjects who were diagnosed between 2010 and 2014 (with at least 1 year of follow up) with BM were incorporated in this study to investigate the effect of female sex on overall survival. Multivariable Cox proportional hazards regression was used after adjusting the aforementioned factors and surgery on the primary site for all cancer types.

A meta-analysis was also conducted to combine the results across different cancer types to calculate the pooled effect of female sex on the occurrence of BM and on overall survival for the total population and for cancers of different systems. The pooled prevalence of BM, the pooled effect of female sex on the development of BM and the effect of female sex on the overall survival were combined using DerSimonian and Laird’s random-effects model.

Normally distributed continuous variables were summarized as the mean values ± standard deviations. Categorical variables were presented as counts and percentages. Statistical analyses were performed using SPSS 23.0 (IBM Corporation, Armonk, NY). Meta-analysis was synthesized using Comprehensive Meta Analysis version 2.0 (Biostat, Englewood, NJ, USA). Two-tailed statistically significant levels were set at *P* < .05.

## Results

### Study population

**Fig. 1:**
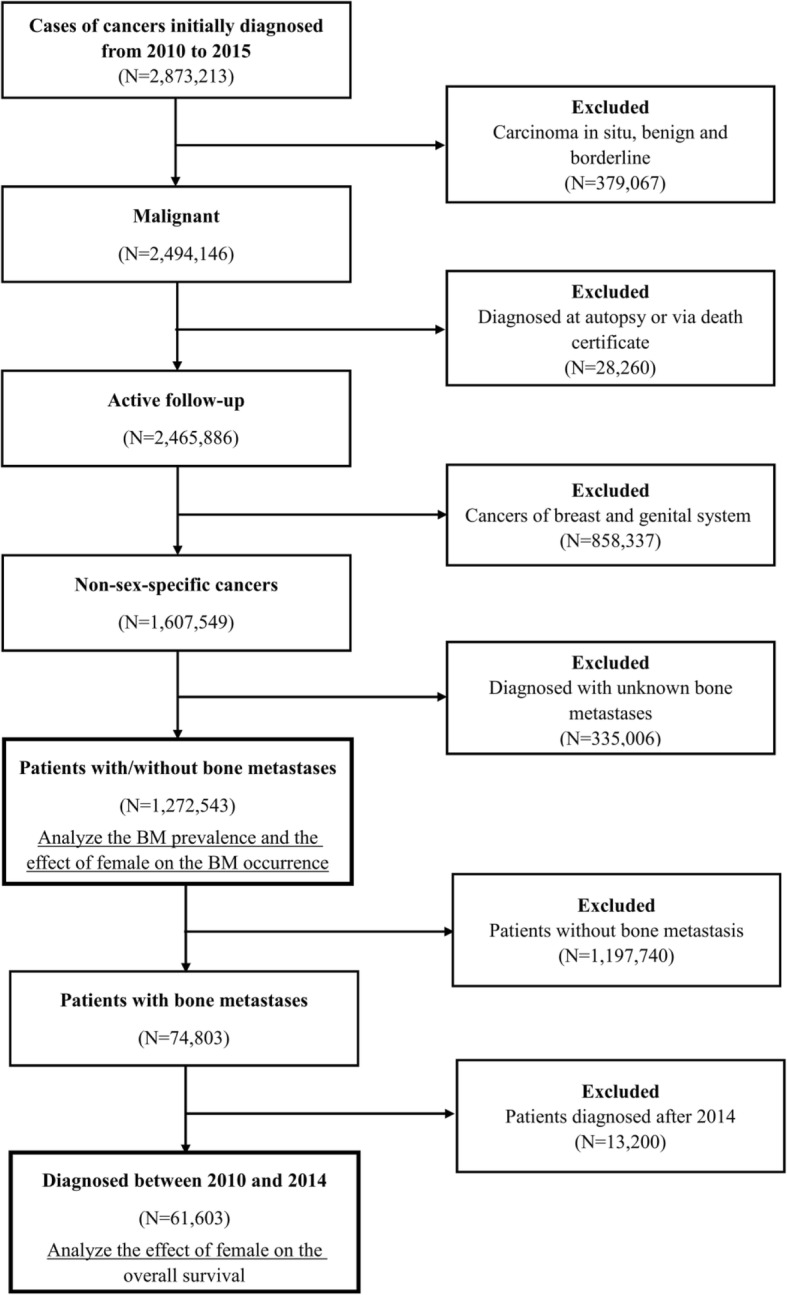
Flow-chart of the non-sex-specific cancer patient selection procedure

A total of 1,272,543 eligible cancer patient profiles were extracted from the SEER database. A flow-chart of the population selection procedure is shown in Fig. 1. A majority of the patients were White (*N* = 1,044,001, 83.0%), followed by Black (*N* = 123,688, 9.8%), Asian or Pacific Islander (*N* = 83,083, 6.6%) and American Indian/Alaska native (*N* = 7812, 0.6%). Female patients accounted for 43.3% (*N* = 550,718) of the total population. The mean age of all patients was 65.23 ± 15.21 years old (65.73 ± 14.23 years for males and 64.57 ± 16.38 years for females). Approximately 56.8% of the cancer patients were married (*N* = 663,632), and 80.7% (*N* = 1,027,545) of them were covered by insurance or other medical aid.

### Prevalence of bone metastases

**Fig. 2:**
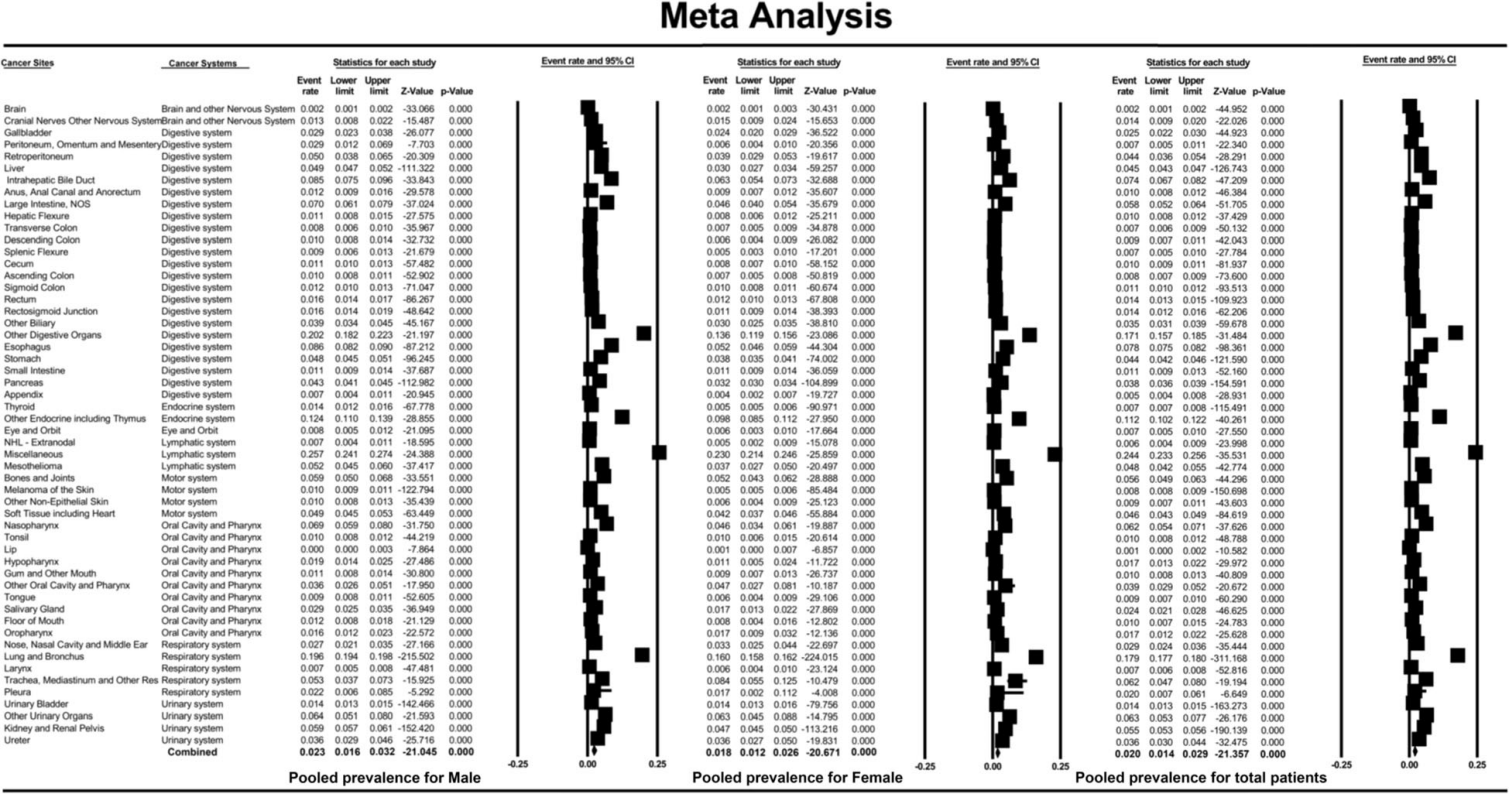
Forest plot for the prevalence of bone metastases across different non-sex-specific cancer types and the pooled prevalence of bone metastases in male patients, female patients and the total population. (For detail information of the figure, please see https://pan.baidu.com/s/1VU4VpV7w90S9k-FHuKS8LQ, password: p55j)

A total of 74,803 patients were diagnosed with BM at diagnosis [*N* = 45,091 (60.3%) for males and *N* = 29,712 (39.7%) for females]. The prevalence of BM for different cancer types ranged from 0.2% (95% CI: 0.1–0.3%, brain) to 24.4% (95% CI: 23.3–25.6%, miscellaneous). The prevalence of BM in male and female patients showed similar results (Fig. 2). Meta-analysis showed that the pooled prevalence of BM for the total population, male patients and female patients was 2.0% (95% CI: 1.4–2.9%), 2.3% (95% CI: 1.6–3.2%) and 1.8% (95% CI: 1.2–2.6%), respectively.

**Fig. 3:**
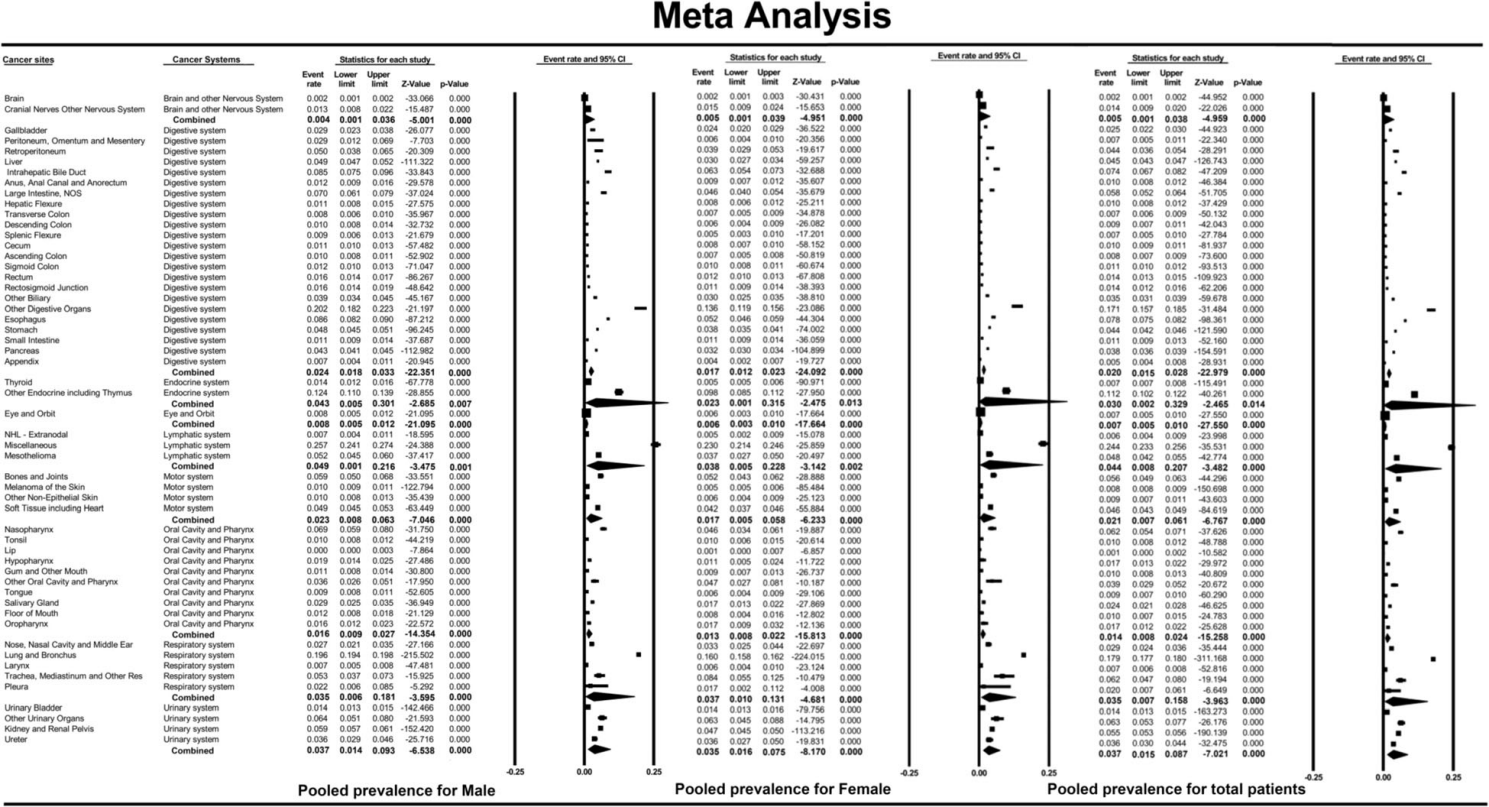
Forest plot for the pooled prevalence of bone metastases for different non-sex-specific cancer systems in male patients, female patients and the total population. (For detail information of the figure, please see https://pan.baidu.com/s/1VU4VpV7w90S9k-FHuKS8LQ, password: p55j)

Different cancers showed an inconsistent prevalence of BM. The lymphatic system exhibited the highest pooled prevalence of BM (4.4%; 95% CI: 0.8–20.7%), followed by the urinary system (3.7%; 95% CI: 1.5–8.7%), the respiratory system (3.5%; 95% CI: 0.7–15.8%), the endocrine system (3.0%; 95% CI: 0.2–32.9%), the motor system (2.1%; 95% CI: 0.7–6.1%), the digestive system (2.0%; 95% CI: 0.15–2.8%), the oral cavity and pharynx (1.4%; 95% CI: 0.8–2.4%) and the eye and orbit (0.7%; 95% CI: 0.5–1.0%). The brain and other parts of the nervous system demonstrated the lowest prevalence of BM (0.2%; 95% CI: 0.1–0.2%). The results for the male and female patients showed similar results (Fig. 3).

**Fig. 4:**
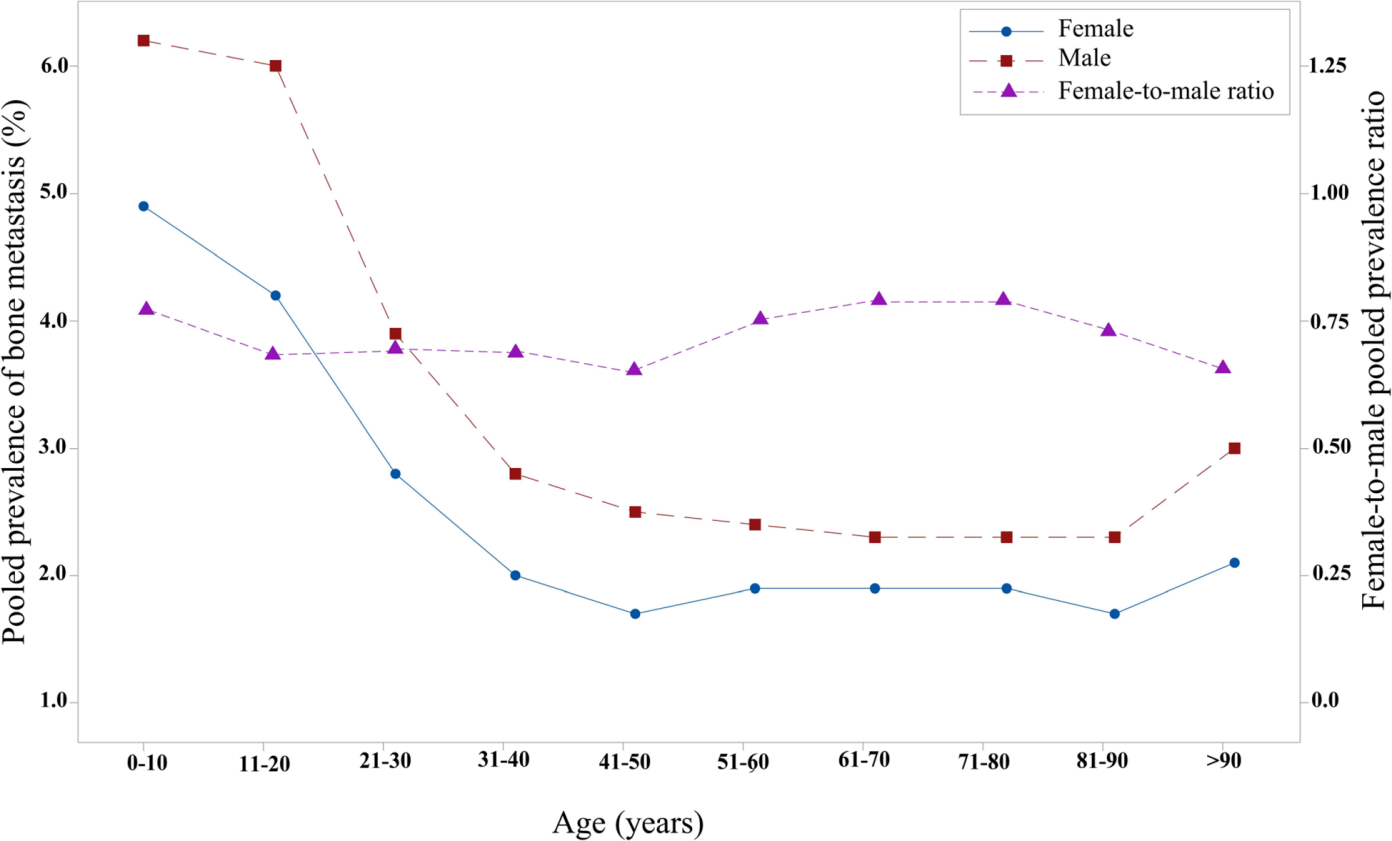
The pooled female and male prevalence of bone metastases and the female-to-male prevalence ratio across different age groups

The prevalence of BM in male and female patients demonstrated marked fluctuations with age. As age increased from 0 to 40 years old, the pooled prevalence of BM of male patients and female patients markedly decreased, and then the pooled prevalence of BM plateaued from 41 to 90 years old. However, for the group with age > 90 years, the combined prevalence of BM for both male and female patients notably increased. Moreover, the results suggested that the female patients had a lower prevalence of BM than male patients, and the female-to-male prevalence ratio was constant across all age groups (Fig. 4).

### Effect of female sex on bone metastases

**Fig. 5:**
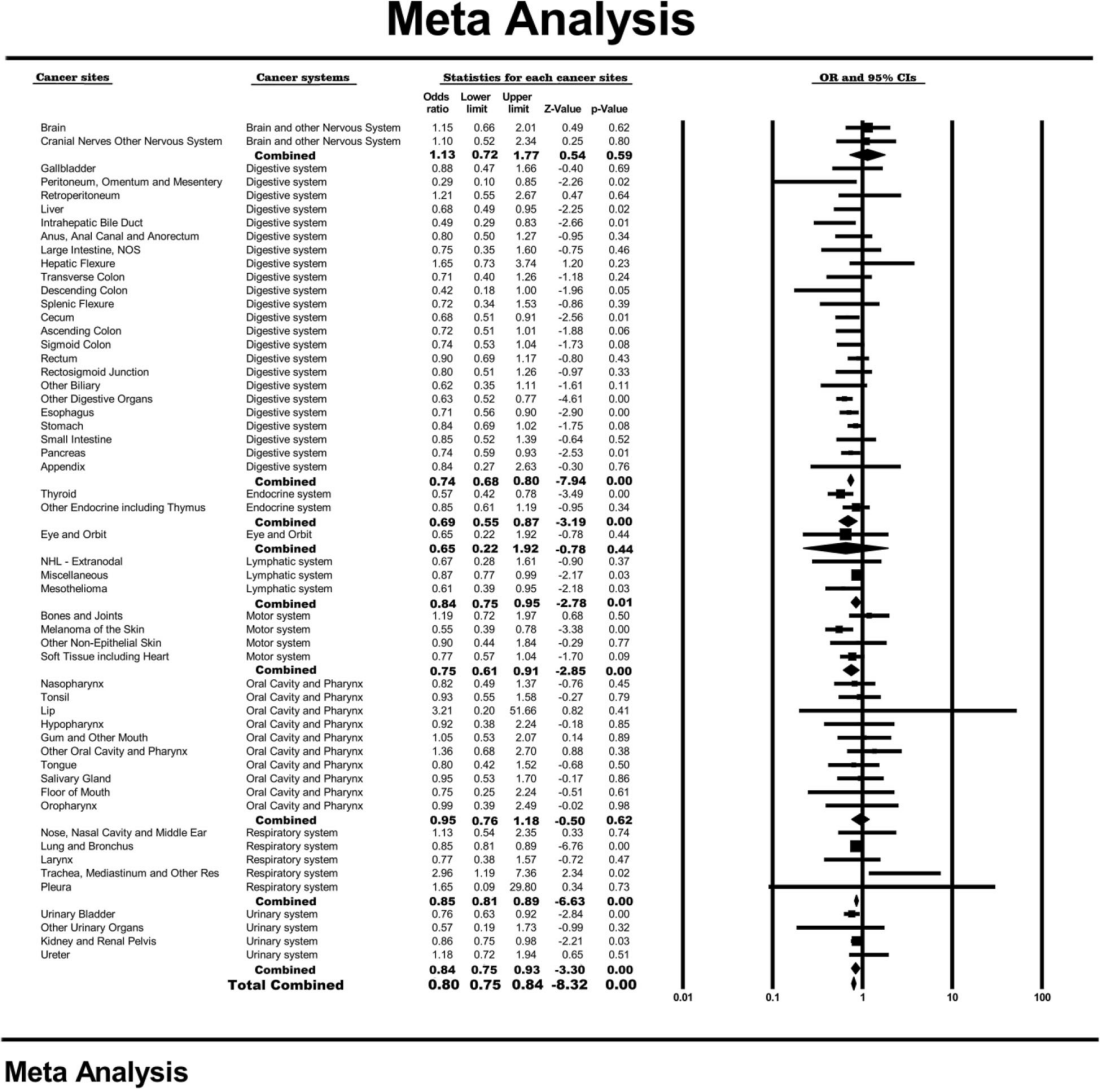
Forest plot for the effect of female sex on the development of bone metastases across different non-sex-specific cancer types and the pooled effect for different non-sex-specific cancer systems and the total population. (For detail information of the figure, please see https://pan.baidu.com/s/1VU4VpV7w90S9k-FHuKS8LQ, password: p55j)

A multivariate logistic regression model showed that the effect of female sex on BM was inconsistent among different cancer types. Female sex exerted the strongest protective effect on the peritoneum, omentum and mesentery (OR = 0.29, 95% CI: 0.10–0.85, *P* = 0.02), followed by the descending colon (OR = 0.42, 95% CI: 0.18–1.00, *P* = 0.05) and the intrahepatic bile duct (OR = 0.49, 95% CI: 0.29–0.83, *P* = 0.01). However, female sex was positively associated with the development of BM in the trachea, mediastinum and other cancer types (OR = 2.96, 95% CI: 1.19–7.36, *P* = 0.02). Meta-analysis, after combining all the cancer types, showed that the female patients had a lower risk of BM than the male patients (pooled OR = 0.80; 95% CI: 0.75–0.84; *P* < 0.001) (Fig. 5).

Meta-analysis of the cancers in different systems showed that female sex had a protective effect with respect to the occurrence of BM for the endocrine system (OR = 0.69, 95% CI: 0.55–0.87, *P* < 0.001), the digestive system (OR = 0.74, 95% CI: 0.68–0.80, *P* < 0.001), the motor system (OR = 0.75, 95% CI: 0.61–0.91, *P* < 0.01), the lymphatic system (OR = 0.84, 95% CI: 0.75–0.95, *P* = 0.01), the urinary system (OR = 0.84, 95% CI: 0.75–0.93, *P* < 0.01) and the respiratory system (OR = 0.85, 95% CI: 0.81–0.89, *P* < 0.01). However, no significant association was found for the brain and other parts of the nervous system (OR = 1.13, 95% CI: 0.72–1.77, *P* = 0.59), for the eye and orbit (OR = 0.65, 95% CI: 0.22–1.92, *P* = 0.44) or for the oral cavity and pharynx system (OR = 0.95, 95% CI: 0.76–1.18, *P* = 0.62).

### Effect of female sex on overall survival in patients with bone metastases

**Fig. 6:**
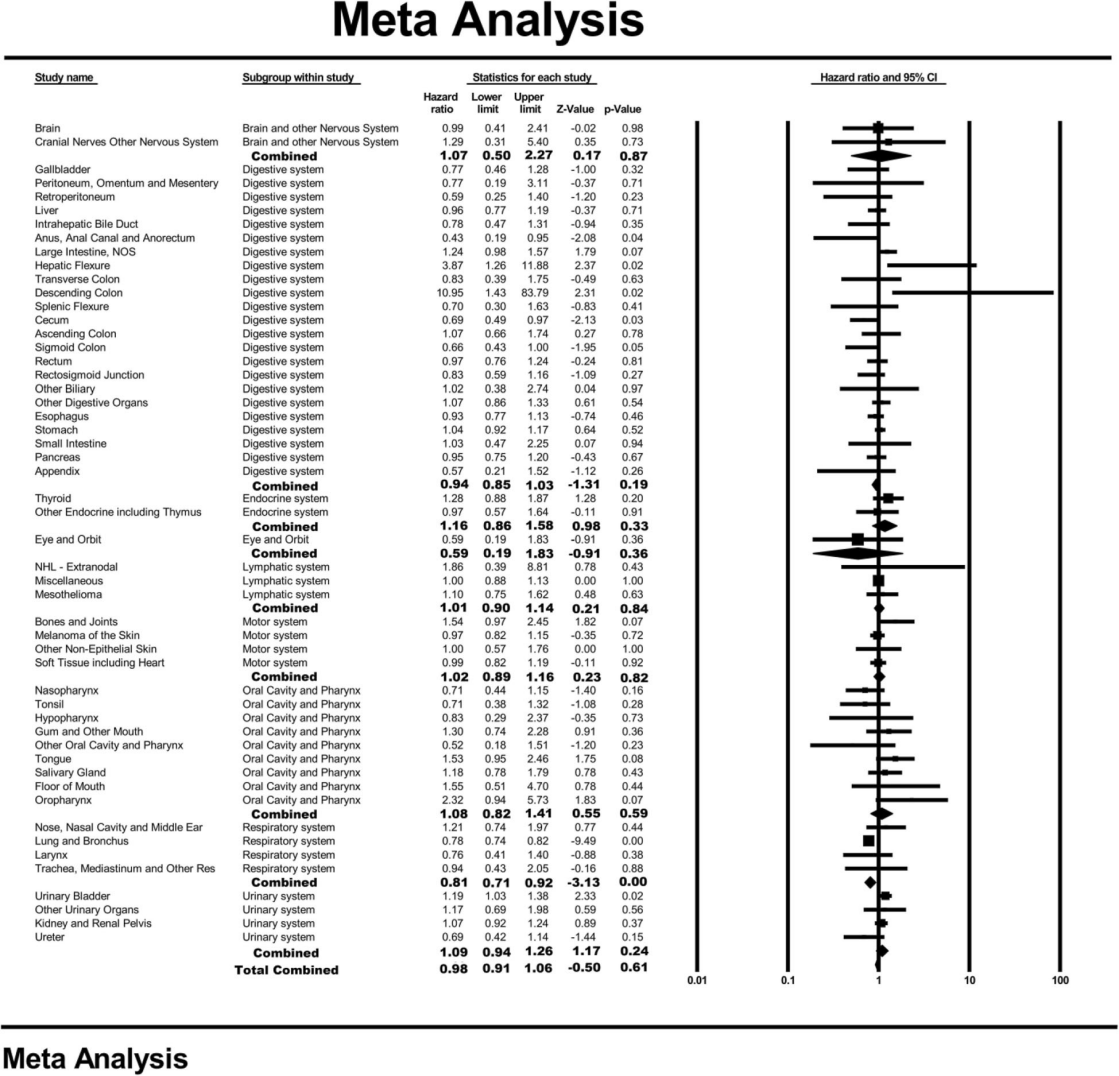
Forest plot for the effect of female sex on the prognosis of bone metastases across different non-sex-specific cancer types and the pooled effect for cancers of different systems and the total population. (For detail information of the figure, please see https://pan.baidu.com/s/1VU4VpV7w90S9k-FHuKS8LQ, password: p55j)

Among the patients enrolled in this study, 61,603 patients with BM (60.4% males and 39.6% females) were included in an analysis to investigate the effect of female sex on the prognosis of patients with BM. The mean age of the participants was 66.67 ± 13.17 years (66.36 ± 12.92 years for males and 67.14 ± 13.53 years for females). The mean follow-up of the participants was 7.65 ± 10.40 months, and the 1-year and 3-year overall survival rates were 21 and 6%, respectively. Multivariable Cox regression showed that the hazard ratio of female sex for overall survival in different cancer types ranged from 0.43 (95% CI: 0.19–0.95; *P* = 0.04; anus, anal canal and anorectum) to 10.95 (95% CI: 1.43–83.79; *P* = 0.02, descending colon). The pooled effect across all cancer types was 0.98 (95% CI: 0.91–1.06; *P* = 0.61) (Fig. 6).

When stratified by different systems, female sex had a protective effect on overall mortality in cancers of the respiratory system (pooled HR = 0.81; 95% CI: 0.71–0.92; *P* < 0.001). However, no significant association was found for the other systems (Fig. 6). When excluding the patients with respiratory system cancer, the pooled effect of female sex on overall survival in other cancer types was 1.00 (95% CI: 0.94–1.07; *P* = 0.93).

## Discussion

To our knowledge, the present study was the first to systematically examine the disparity of the occurrence and prognosis of BM among the sexes in millions of patients with non-sex-specific cancers. The prevalence of BM in different cancers ranged from 0.2% (brain) to 24.4% (miscellaneous), and the pooled prevalence of BM for all patients was 2.0% (95% CI: 1.4–2.9%). The current study showed that the trend in the prevalence of BM dramatically decreased for ages between 11 and 40 years old, plateaued for ages between 41 and 90 years old and increased for ages > 90 years old in both male and female patients.

On the other hand, the present study showed that female patients had a lower prevalence of BM than male patients, and the female-to-male pooled prevalence ratio was consistent across different age groups. Compared with male sex, female sex had a protective effect on the development of BM as suggested by meta-analysis. A series of clinical studies suggested that the occurrence of BM was more prevalent in male patients, which was in line with our results [18, 21]. One of the potential explanations for the results may be that sex hormones have different influences on the development of BM.

Sex hormones have been accepted as key factors in musculoskeletal health for both males and females [24]. Both androgens and oestrogens can affect the proliferation of osteoblasts and osteoclasts. In both males and females, oestrogens were reported to directly inhibit osteoclasts and exert an effect on the maintenance of bone mass [24, 25]. Moreover, androgens are thought to contribute directly to male periosteal bone expansion, mineralization, and trabecular bone maintenance, which are important in the pathogenesis of BM [26, 27]. The latest review suggested that hormonal status affected the occurrence of BM [28]. In our study, the relationship between the occurrence of BM and age was in contrast with the tendency of human sex hormone levels to change with age [29]. Thus, we proposed the hypothesis that female hormones play a significant protective role in regulating BM in non-sex-specific cancer patients. Oestrogens were reported to be able to reverse the inhibitory effects of osteoblasts on osteogenic differentiation through regulation of the RANKL–osteoprotegerin pathway [30, 31], which further supports our hypothesis.

In the present study, compared with male sex, female sex was a favourable prognostic factor for patients with respiratory system cancers, which was consistent with the results of previous studies [32, 33]. Positive expression of the hormonal receptor such as ER-α was reported to be one of the favourable prognostic factors for lung cancer patients. Furthermore, increased ER-α expression was reported in the lung tissue of female patients [23, 34], which may explain our results indicating that female sex is a favourable prognostic factor for respiratory system cancer patients. At the same time, a previous report suggested that the better prognosis of female lung cancer patients could also be attributed to the better response to EGFR inhibitor treatment for females [35, 36].

The aforementioned findings suggest that male cancer patients and those with male-leaning genetic variations or hormonal status have higher odds of developing BM. Second, cancer patients with high male sex hormone levels at initial diagnosis can potentially be selected as candidates for screening for BM. Third, hormone therapy could be a potential therapeutic strategy for non-sex-specific cancer patients with BM. More studies will be needed to investigate the mechanism of the protective effect of sex hormones on the development and prognosis of BM. The use of hormonotherapy as a treatment option for non-sex-specific cancer should also be confirmed in biological research and clinical trials. All the possible findings may improve the prevention, screening and treatment of BM in patients with cancer.

There are some limitations in the present study. First, the diagnostic approach for initial BM among cancer patients was not recorded by the SEER database, and the asymptomatic cases and the patients who developed BM later during the disease course were not recorded in the database. Accordingly, the prevalence of BM may have been underestimated to some extent, and more studies are needed to further confirm the results. Second, there were significant heterogeneities in the meta-analyses. Although the random effect model could partly solve the problem, the combined results were relatively conservative, and the results should be interpreted with caution. In addition, skeletal-related events were not recorded in the SEER database, resulting in difficulty in evaluating their influence on survival and quality of life. All these weaknesses should be improved upon in future studies.

## Conclusions

In summary, the prevalence of BM in both sexes inversely fluctuated with changes in age. Among non-sex-specific cancer patients, compared with male sex, female sex was associated with less frequent development of BM, and the trend was consistent with changes in age. Female sex exclusively showed a protective effect on the prognosis of respiratory system cancers. All the findings in the present study will provide useful guidelines for screening for BM and prediction of survival. More studies should be conducted to investigate the underlying mechanisms of hormone disparities and to discover more effective treatment methods from the view of hormone disparities.

## Data Availability

The data were extracted from the Surveillance, Epidemiology, and End Results (SEER) database. This is an open database (https://seer.cancer.gov).

## Abbreviations

BM: Bone metastases
SEER: Surveillance, Epidemiology, and End Results
SREs: Skeletal-related events
